# High frequency of carbapenem-resistant Enterobacteriaceae fecal carriage among ICU hospitalized patients from Southern Iran

**DOI:** 10.22038/IJBMS.2022.63099.13938

**Published:** 2022-12

**Authors:** Nima Davari, Reza Khashei, Bahman Pourabbas, Vajihe Sadat Nikbin, Farid Zand

**Affiliations:** 1 Department of Bacteriology and Virology, School of Medicine, Shiraz University of Medical Sciences, Shiraz, Iran; 2 Professor Alborzi Clinical Microbiology Research Center, Shiraz University of Medical Sciences, Shiraz, Iran; 3 Department of Bacteriology, Pasteur Institute of Iran, Tehran, Iran; 4 Anesthesiology and Critical Care Research Center, Shiraz University of Medical Sciences, Shiraz, Iran

**Keywords:** Antibiotic resistance, Carbapenemase, Enterobacteriaceae, Fecal carrier, PFGE

## Abstract

**Objective(s):**

The worldwide emergence of carbapenem-resistant Enterobacteriaceae (CRE) has become a major therapeutic concern to medical institutions. To date, no study has determined the frequency and risk factors of inpatients with CRE fecal carriage in Southern Iran. We studied the features of carbapenemase-producing Enterobacteriaceae (CPE) collected from the central ICU of a university hospital.

**Materials and Methods:**

Totally, 173 samples, including 124 stool samples from 46 ICU inpatients on admission and different follow-ups, 9 ICU staff, and 40 environmental samples were included. CRE was identified using microbiological methods. Antimicrobial susceptibility was investigated by using the disk diffusion method and E-test. Carbapenemase producers were detected using the mCIM method. Seven carbapenemase genes were characterized. The genetic relationship among 20 CPE was elucidated by PFGE.

**Results:**

The overall fecal carriage rate was 28.2%, while CRE acquisition was 6.1%. CRE were classified as *Klebsiella pneumoniae *(71.4%), *Escherichia coli* (23.8%), and *Enterobacter aerogenes* (4.8%). From 21 CRE, 20 (95.2%) produced carbapenemases, of which 10, 15, 10, 25, 5, and 65% were *blaKPC*, *blaSME*, *blaIMP*, *blaVIM*, *blaNDM* and *blaOXA-*48-positive, respectively. Out of 20 CPE, 14 different PFGE patterns were observed, categorized into six clusters, suggestive of non-clonal spread. No difference between the examined risk factors with CRE carriage was shown.

**Conclusion:**

The data indicate a high CRE fecal carriage rate among inpatients. Our findings implicate the widespread of OXA-48 carbapenemase together with heterogeneity among CRE with great concern for dissemination and therapeutic threat. Early diagnosis and monitoring of CRE among inpatients are urgent.

## Introduction

Enterobacteriaceae members as Gram-negative bacteria (GNB) can cause a variety of community- and healthcare-associated infections such as soft tissue, bloodstream, respiratory tract, and urinary tract infections (1, 2). However, multidrug-resistant (MDR)-GNB are notably described in patient care units, and carbapenems are regarded to be one of the last treatment options for severe infections due to MDR-GNB (2-4). Nonetheless, overuse of carbapenems in medical practice resulted in the emergence of carbapenem-resistant Enterobacteriaceae (CRE) around the world (2, 5). Recently, CRE have been distributed globally and are mainly related to the production of carbapenemases, leading to resistance of GNB to most β-lactams, including carbapenems. This issue has limited treatment options with high mortality (>50%), which poses challenges in hospital settings (6, 7). According to the Ambler scheme, various carbapenemases, including KPC, GES, SME, IMP, VIM, NDM, and OXA-48, have been involved in carbapenem resistance as the most common mechanism and reported with different prevalence’s globally (8, 9).

Since Enterobacteriaceae constitute one of the most important intestine microflora, everyone can unknowingly become a CRE reservoir. This fecal carriage is an important concern for the infection control practitioner because the gut can be considered a reservoir for exchanging resistance genes among bacteria, in particular MDR-GNB. As a strategy to control cross-transmission, screening of CRE intestinal carriage is used by many hospitals, especially in intensive care units (ICUs). Early diagnosis of CRE carriers allows the implementation of contact precautions to prevent the dissemination of CRE to other inpatients (10, 11).

CRE may contaminate ICU staff hands, gloves or gowns, and environmental surfaces surrounding patients, providing potency for further spread among inpatients and personnel. Inpatients in ICU wards are usually at risk of developing nosocomial CRE with high morbidity and mortality and poor outcomes (12). Suggested risk factors for CRE carriage are using enteral feeding tubes, improper use of antibiotics, length of hospitalization, and clinical characteristics of patients (10, 13). Until now, little information has been available on CRE fecal carriage in Iran. The aim of this study was to gain insight into the microbiological features and molecular epidemiology of CRE isolated from ICU patients and staff in a tertiary hospital from Shiraz, south of Iran. 

## Materials and Methods

***Patient samples and data collection***

This cross-sectional study was conducted from July to November 2020 in the central ICU of Shiraz Namazi Teaching Hospital with 20-bed units. During the study period, 124 stool samples or rectal swabs from 46 sequential patients were recruited. Thirty-three patients were followed-up, and the rest of the patients were discharged or transferred to another ward or expired. The samples were taken in the first 24 hr after admission to the ICU ward. Indeed, on the third and seventh days after admission, patients who were still hospitalized in the ICU were eligible for determining CRE acquisition with stool culture until the patient was discharged, died, or transferred to other wards. On admission, a written informed consent and questionnaire form containing demographic data (e.g., gender, age, and other background information) and possible risk factors (e.g., previous hospitalization, surgery in the last year, antibiotic use during the last month, the presence of invasive devices, indwelling catheters, mechanical ventilation, autoimmune disease and receiving hemodialysis or chemotherapy) associated with CRE intestinal colonization was obtained from all participants. Moreover, 9 stool specimens and 40 environmental samples were collected from ICU staff (nurses and environmental cleaners) and patients’ beds comprising bed linen around the pillow, bed rails, bedside table, cardiovascular monitor screen, oxygen mask, suction machine, and ventilator machine buttons, respectively. This study followed the declaration of Helsinki and was approved by the Ethics Committee of Shiraz University of Medical Sciences (approval number IR.SUMS.REC.1399.467).

***Culture of stool samples and identification of isolates***

After collecting specimens and transportation to the laboratory, approximately 0.5 gr of stool samples were emulsified in 1 ml of sterile 0.9% saline, and 10 μL of the resulting suspension was plated onto MacConkey agar (Que Lab Co. UK). A 10-µg ertapenem disk (MAST Co., UK) was then placed as described previously (14). The plates were incubated overnight at 37 °C. An inhibition zone ≤ 27 mm around the ertapenem disk was defined as ertapenem-resistant Enterobacteriaceae isolates or CRE, as reported by Lolans *et al. *(14). All distinct colony types were identified by standard microbiological tests and confirmed to species level using API 20E kit (bioMérieux, Marcy l’Etoile, France). Moreover, for phenotypic confirmation of CRE isolates, the presumptive colonies were streaked on CHROMagar KPC (CHROMagar Company, Paris, France) medium and, after overnight incubation at 37 °C were investigated according to manufacturer’s instructions (*Klebsiella* and *Enterobacter* species, medium-size dark metallic blue colonies; *Escherichia coli*, medium to large pink/dark rose colonies) (15, 16). All isolates were stored at -70 °C in tryptic soy broth (Merck Co., Germany) containing 20% glycerol before investigation. 

***Susceptibility testing***

All CRE isolates were tested on Mueller-Hinton agar plates (Quelab Co. UK) for resistance to ampicillin, aztreonam, meropenem, imipenem, ciprofloxacin, ceftazidime, cefoxitin, amikacin, piperacillin-tazobactam and gentamicin by the disk diffusion technique using the criteria published by the Clinical and Laboratory Standards Institute (CLSI) (17). Based on CLSI, resistance to one or more carbapenems is considered carbapenem resistance (18). *E. coli* ATCC 25922 was used as the control strain. MDR was defined as non-susceptibility to ≥1 agent in ≥3 different antibiotic classes (19). Moreover, all isolates with intermediate or resistant to carbapenems (imipenem, meropenem, and ertapenem) were further evaluated by MIC determination using E-test strips (Liofilchem Co., Italy) and interpreted according to CLSI recommendations (17).

***Phenotypic detection of carbapenemase***

Detection of carbapenemase-producing Enterobacteriaceae (CPE) isolates was performed and interpreted by the modified carbapenem inactivation method (mCIM) according to CLSI guidelines (17). Briefly, suspension of bacteria was prepared in 2 ml of tryptic soy broth, and a 10-μg meropenem disk (MAST Co., UK) was added and incubated at 35 °C for 4 hr. Since then, the meropenem disk was placed on Mueller-Hinton agar which was inoculated with a 0.5 McFarland suspension of *Escherichia coli* ATCC 25922 and incubated overnight at 35 °C. The presence of carbapenemase activity was recognized by an inhibition zone of 6-15 mm in diameter or the presence of pinpoint colonies within a 16-18 mm zone, and the absence of carbapenemase activity was revealed by zone diameter of ≥19 mm. 

***Detection of carbapenemase genes***

Bacterial genomic DNA was extracted from CRE isolates using the boiling method (20). PCR was carried out on a thermal cycler (The Applied Biosystems Veriti ™ 96-Well Thermal Cycler, Eppendorf, Germany) using the primers published previously allowed the detection of carbapenemase-encoding genes, including *bla*_KPC_, *bla*_GES_, *bla*_SME_, *bla*_IMP_, *bla*_VIM_, *bla*_NDM_, and *bla*_OXA-48_ (21-25). The electrophoretic separation was performed in a 1.2% agarose gel with Eco-Stain (Bio Basic Inc. Canada) and visualized with UV light by the Gel Doc system.

***Pulsed-ﬁeld gel electrophoresis (PFGE) genotyping***

PFGE was conducted to conﬁrm the clonal relationship among CPE (n=20) recovered from ICU patients and environment. Restriction enzyme *XbaI*-digested (Fermentas, Canada) genomic DNAs were prepared at 37 °C for 12–14h. The *XbaI*-digested DNA of *Salmonella* Braenderup strain H9812 was used as a control strain and molecular weight marker. DNA fragments were separated using a CHEF-DRIII System (Bio-Rad Laboratories, Hercules, CA, USA). Electrophoresis was performed for 18 h at 14 °C with pulse time ranging from 6.76 to 35.38 sec at 6 V/cm. NA agarose gels (1%) were then stained with a safe stain. Banding patterns were analyzed with GelCompar II software v6.6.11 (Applied Maths NV, a bioMérieux Co., Belgium) to generate a dendrogram. Pulsotypes were clustered through Dice correlation coefficients with optimization of 1% and tolerance of 1%. The isolates were considered genetically related if the Dice coefficient of correlation was ≥ 80% (26). 

***DNA sequence analysis***

The amplicons were submitted for sequencing (Bioneer Co., Munpyeongseoro, Daedeok-gu, Daejeon, South Korea), and the sequences were compared using online BLAST software (http://www.ncbi.nlm.nih.gov/BLAST/) to confirm the accuracy of amplified carbapenemase genes (one sample of each positive gene).

***Statistical data analysis***

The analysis was done using SPSS™ software, version 26.0 (IBM Corp., USA). Chi-square, Fisher’s exact test, and 2-tailed *t*-test were used to correlate participants’ demographics data with CRE intestinal carriage, and differences were regarded statistically significant when the *P*-value was ≤ 0.05.

## Results

***Study population and clinical characteristics of CRE isolates***

Our population included 173 stool samples or rectal swabs from 124 specimens (comprising 46 patients in different follow-ups) and 9 staff of the central ICU, including 31 (56.3%) men and 24 (43.7%) women with a median age of 60 years (range=25 to 95 years) and 40 environmental samples. In all, 21 (12.1%) CRE isolates were recovered and confirmed. These isolates comprised 20 strains from 13 inpatients at admission as fecal carriage, their follow-up as CRE acquisition, and one environmental sample. The frequency of CRE intestinal colonization on admission to the ICU ward was 28.2% (13 isolates from 46 inpatients). Of the 20 isolates, 4 were obtained from followed-up rectal swabs or stool samples on the third and seventh days after admission. In other words, two patients acquired CRE (4 *K. pneumoniae*) after admission as CRE acquisition or nosocomial infection with a frequency of 6.1%. In addition, 4 isolates were recovered from two patients on admission and their follow-ups; however, the isolates were the same (*K. pneumoniae*) (Table 1). All of these isolates were carbapenemase producers, except one isolate recovered from a patient at admission (*K. pneumoniae*). None of the 9 personnel carried CRE isolates in their stools. The only environmental specimen was identified as *K. pneumoniae*. All CRE were classified as *K. pneumoniae* (n=15, 71.4%), *E. coli* (n=5, 23.8%), and *Enterobacter aerogenes* (n=1, 4.8%). All 46 patients had variable underlying conditions, such as malignancy, diabetes mellitus, and respiratory disease (Table 2). In addition, 14 patients had undergone surgery within 1-3 months. Twelve CRE isolates were recovered from patients who received antibiotics within 3 months ago. Unfortunately, 4 patients with CRE-positive culture died in the hospital. There was no significant difference between the examined risk factors with CRE fecal carriage (Table 2).

***Antimicrobial resistance among CRE isolates***

The results of susceptibility testing are depicted in Table 3. All 21 clinical isolates revealed resistance to most antimicrobial agents with different proportions. Of all the tested antibiotics, the most resistant antimicrobial was ertapenem (100%), aztreonam (100%), and ceftazidime (100%). Conversely, the lowest resistance rate was against piperacillin-tazobactam (57.1%). Among CRE, *K. pneumoniae* represented the highest (93.3%) resistance to antimicrobial agents (Table 4). All isolates (n=21, 100%) exhibited MDR phenotype. Among 21 carbapenem-resistant isolates, 20 (95.2%) showed positive results with mCIM test and were regarded as CPE. All of the mCIM-positive isolates were MDR. Sixteen (%76) isolates were phenotypically not susceptible to all carbapenems, according to CLSI. Indeed, 76, 100, and 90% of CRE isolates were not susceptible to imipenem, ertapenem, and meropenem using the E-test, respectively (CLSI breakpoints: ≤ 1 mg/l for IMP and MEP: susceptible, and ≥ 4 mg/l: resistant; ≤ 0.5 mg/l for ETP: susceptible, and ≥ 2 mg/l: resistant). Three *E. coli* isolates were susceptible to imipenem with MIC values of 0.25, 0.25, and 0.3 mg/l but resistant to other carbapenems. One *K. pneumoniae* isolate was susceptible to both imipenem and meropenem with MICs 0.25 and 0.032 mg/l, respectively, but resistant to ertapenem, and finally, one *E. aerogenes* isolate was susceptible to imipenem and meropenem with MICs 0.75 and 0.125 mg/l, respectively, but resistant to ertapenem.

***Characterization of carbapenemase genes***

Carbapenemase genes among 20 CPE had different distributions. In our specimens, 2 (10%), 3 (15%) and 0 (0%) isolates were carried *bla*_KPC_, *bla*_SME_ and* bla*_GES_ as class A carbapenemase, 2 (10%), 5 (25%) and 1 (5%) carried *bla*_IMP_,* bla*_VIM_ and *bla*_NDM_ as class B carbapenemase and 13 (65%) harbored *bla*_OXA-48_, as the dominant type of class D carbapenemase (Table 5). Of note, none of the investigated genes was found in only CRE isolate that was carbapenemase negative with mCIM method. Four strains contained two carbapenemase genes from different classes. Three isolates were positive for *bla*_OXA-48 _and *bla*_VIM_ genes, and one isolate co-harbored *bla*_OXA-48 _and *bla*_SME_ genes. The only CPE isolated from the environmental sample (patient’s bed) harbored *bla*_KPC_ and *bla*_SME_ genes. All 13 *bla*_OXA-48_-positive isolates (100%) were resistant to ceftazidime, aztreonam, and ertapenem. 

***Clonality analysis of isolates***

**Figure 1:**
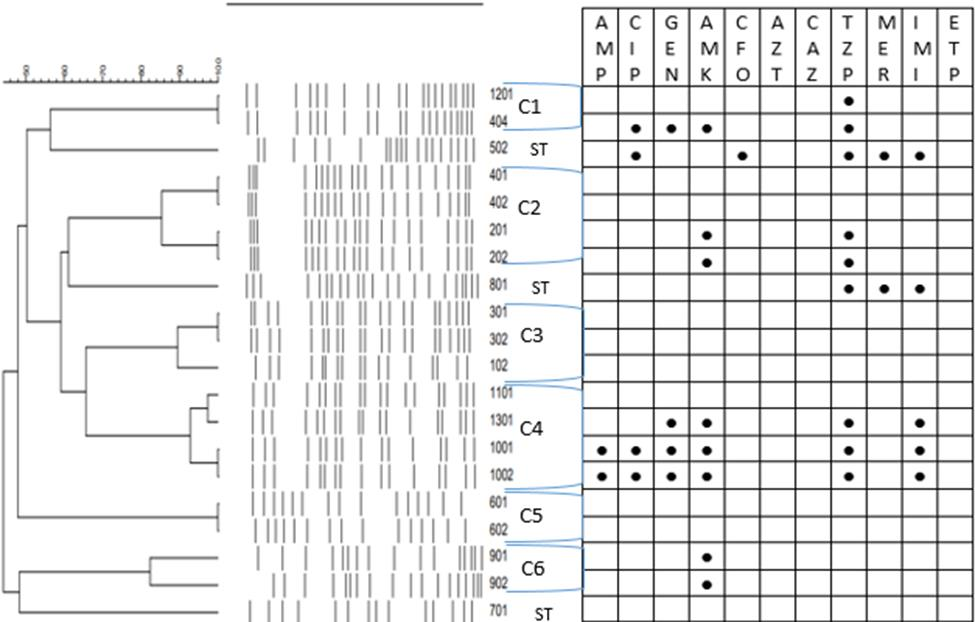
Dendrogram analysis of XbaI-treated pulsed-field gel electrophoresis profile for CRE isolates according to their antimicrobial susceptibility pattern. Blank and black symbols indicate "resistant" and "susceptible", respectively. Strains were considered to be genetically related if the Dice coefficient of correlation was 80% or greater

PFGE analysis revealed a high degree of genetic diversity because 14 different PFGE patterns were obtained from the 20 investigated CPE, which were divided into 6 clusters, while 3 isolates appeared to be single types or singletons (ST) with a genetic linkage of 80% (Figure 1). The STs were 1 (no. 502), 2 (no. 801), and 3 (no. 701). In fact, because of the absence of the dominant strain; 240, there was no common type. Seventeen isolates had the same PFGE patterns but were different from other clusters, and the remaining 3 isolates had their unique patterns. Based on a cutoff of 80% genetic similarity, PFGE categorized our isolates into 6 clusters, including: C1: (1201, 404); C2: (401,402,201,202); C3: (301, 302, 102); C4: (1101, 1301, 1001, 1002); C5: (601, 602); C6: (901, 902). According to this category, Clusters 2 and 4 have the most strains. 

Although samples 901 and 902 were obtained from the same patient on admission and on the third day of hospitalization; however, the two isolates had a similarity of approximately 82%. The environmental specimen (no. 404), isolated from the patient’s bed of number 401 (401 and 402 were isolates recovered from the same patient on admission and his follow-up), had a more genetic relationship with specimen 1201, implying that this sample has been probably transferred by ICU personnel from the patient 1201 to inpatient 401.

## Discussion

For CRE surveillance and infection control interventions, microbiological and molecular characterization of these isolates is necessary in different parts of the world (27). In the present study, the characteristics of CRE collected from asymptomatic carriers in a referral university hospital were investigated by determining carbapenemase production, genes encoding carbapenemase, and typing of isolates using the PFGE. 

The CRE carriage rate varies with different geographic regions, institutions, and times of the studies (2, 5, 6, 28). However, data concerning CRE intestinal colonization in hospitalized patients, particularly among ICU staff and inpatients in Iran, is still rare (29). In our study, as many as 28.2% of patients attending the central ICU were positive for CRE fecal carriage by culture. In addition to stool samples as the gold-standard specimen, rectal swabs for quantifying CRE are also suitable due to ease of collection and processing (30, 31). Our frequency is much higher than those reported from the United States (1.4-4.2%), and many Asian countries (<1-4.05%) (8), and the studies from China (6.6%), Brazil (6.8%), Korea (0.3%), Pakistan (18.3%), Japan (12.2%) and Morocco (13%) (6, 11, 13, 32-34). However, this rate is somewhat similar to, although lower than, that mentioned by Solgi and co-workers, with a rate of 37.9% in Iran (29). On the other hand, the acquisition rate was determined to be 6.1% (2 patients from 33 re-screened patients), which was higher than the study of Kim *et al*. (13) but less than those reported by Salomao and colleagues (11).

The most frequently encountered species were *K. pneumoniae*, *E. coli*, and *E. aerogenes*. However, the most frequently isolated CRE in the United States and European countries are* K. pneumoniae* followed by *E. aerogenes* and *E. coli* (8, 27, 35). Likewise, this trend was observed in China, Taiwan, and Iran as Asian countries (2, 36, 37).

CRE are an imminent threat to ICU patients and can transfer between patients, ICU personnel, and the environment (12). Therefore, screening should be performed for these populations as high-risk patients (5). In an investigation from the UK, CRE contamination in the patients’ bed areas in different wards was shown (38). In our survey, only one CRE isolate was obtained from the bed area of one of the participants. These findings highlight the importance of intervention actions, including surveillance, standard precautions, hand washing, and environmental cleaning in CRE dissemination and transmission to other patients and staff. 

MDR bacteria have become a global concern recently (28, 39). The frequency of our MDR isolates (100%) was much higher than those observed by previous studies (8, 33). Consistent with our survey, the majority of CRE strains obtained from Moscow, Russia, were MDR (40). In the current work, the most active antibiotics against MDR CRE isolates were piperacillin/tazobactam and amikacin, with susceptibility rates of 42.9 and 38.1%, respectively. This suggests that piperacillin/tazobactam could be considered as a last-line for treatment. Similar to Lee *et al*. report (9), the MIC values of CRE isolates revealed that all CRE isolates were not susceptible to at least one of the carbapenems, comprising imipenem, meropenem, and ertapenem. 

In our work, 95.2% of isolates were CPE. In four studies conducted in China, the USA, and Iran (2, 27, 29, 37), carbapenemases were detected in 80, 81.7, 87, and 87% of CRE clinical isolates, respectively, which was compatible with our findings. The remaining isolate was a non-carbapenemase producer due to other resistance mechanisms. Interestingly, in a previous study from Korea, none of the CRE recovered from ICU patients were carbapenemase-positive (13). Other researchers have detected that 43 and 13% of CRE isolates from urine samples and rectal swabs were carbapenemase producers, respectively (7, 33). In the present investigation, environmental sampling yielded one CRE (*K. pneumoniae*) isolate. In a study from Turkey, no carbapenem-resistant bacterium was identified from environmental specimens (41). It seems that this issue is possibly due to the performance of appropriate disinfection procedures by healthcare workers. Nevertheless, in a study from China, 7.99 and 3.57% of environmental and ICU staff specimens were *K. pneumoniae* carbapenem-resistant positive, respectively (12).

The molecular epidemiology of the resistance mechanisms varies according to the geographical area. The most important carbapenemases are KPC, VIM, IMP, NDM, and OXA-48 (2, 8), and among CPE isolates, OXA-48 carbapenemase is commonly identified from Mediterranean and Middle East countries (8, 33). OXA-48 was determined in the highest proportion (n=13, 65%) and may represent a considerable drug-resistant mechanism of carbapenemase producers in our area. In three studies from Iran, Turkey, and Lebanon, OXA-48 was also found to be the most prevalent carbapenemase in their CRE isolates, with a prevalence of 42.6, 69 and 48.1%, respectively (29, 41, 42). Our isolates harboring OXA-48 were completely resistant to ceftazidime, aztreonam, and ertapenem. Likewise, high levels of resistance to extended-spectrum cephalosporins and carbapenems were found in studies from Turkey and Lebanon (8, 42). Additionally, similar to previous reports (8, 28, 29, 37) majority of OXA-48 producers were *K. pneumoniae* isolates. Notably, the co-existence of OXA-48 and VIM in three of our isolates is compatible with very few reported studies (35, 43, 44). In contrast, in another study, no strains carried two or more genes encoding carbapenemases (45). 

KPC was found in 2 (14%) of our CRE (*K. pneumoniae*) isolates. Conversely, in studies from Turkey and Iran, no KPC carbapenemase was detected (28, 46). VIM-positive Enterobacteriaceae are found prevalently in Mediterranean countries (8). In the current study, we identified *bla*VIM in 3 *K. pneumoniae* and 2 *E. coli* isolates which also co-harbored the *bla*OXA-48 gene. By contrast, no VIM, KPC, and IMP genes were detected in a survey from Saudi Arabia (7).

Indeed, *bla*NDM was most commonly identified in *K. pneumoniae,* and *E. coli* isolates but has been reported in other Enterobacteriaceae members (8). We found only one NDM-1 producing *E. aerogenes* in our study. Nevertheless, NDM-1 producer *Enterobacter* isolates have also been reported in previous studies from Iran (29, 37, 46). In contrast to our survey, in a study performed on carbapenem-resistant Gram-negative rods, including Enterobacterales recovered from clinical samples, only *bla*NDM and *bla*VIM were detected, and other examined carbapenemase genes were not found (3). Similarly, no GES carbapenemase was reported by Solgi *et al*. and Karaaslan and colleagues among their CRE isolates (37, 41). In agreement with our findings, another study detected SME and NDM-1 genes in 3 (2.6%) and 1 (0.9%) of their clinical isolates, respectively (27).

Regarding one non-carbapenemase producer isolate which was PCR negative for all of our screened genes, it has been cited that this might be due to the presence of BKC-1 carbapenemase (Brazilian *Klebsiella *Carbapenemase), a recently described enzyme, or other resistance mechanisms such as porins defects and production of ESBLs (TEM, SHV, and CTX-M) or AmpC β-lactamases (2, 11). As a result, the distribution of carbapenemase genes varies according to geographical regions, even within a country.

PFGE analysis demonstrated a high degree of genetic heterogeneity because 14 different pulsotypes were obtained from the 20 examined CPE. However, six well-defined clusters, 3 STs, and no CT (common type) with a genetic linkage of 80% were determined. Our results point to a non-clonal spread because the CPE isolates had non-related patterns. The similarity of some PFGE patterns indicates that those isolates were recovered from the same patients on different days. Similarly, the non-clonality among *E. coli* fecal isolates has also been reported by various studies (10, 13, 29). On the contrary, in two other studies, clonal dissemination was diagnosed among 10 *Serratia marcescens* from clinical samples and 64 *K. pneumoniae* fecal isolates (2, 12). 

Moreover, risk factors for intestinal colonization with CRE were determined. It is well known that identifying risk factors for monitoring colonized patients with CRE is helpful, because it might prevent dissemination of them and the development of nosocomial infections (6). However, in the present survey, no risk factor, including age, gender, length of hospital stays, or antimicrobial exposure associated with CRE fecal carriage in the ICU ward was identified. It seems that this may be attributed to the small sample size. Consistent with our results, Zhao *et al*. revealed no significant difference between some variables such as age, sex, length of hospitalization, and use of some antibiotics with CRE fecal carriage (6). In another work from the USA, none of the studied risk factors were associated with the carriage of carbapenem-resistant Gram-negative bacteria (47). On the other hand, however, several studies have shown the association of some risk factors, including length of hospitalization, antibiotic exposure and other variables, with CRE fecal carriage (1, 10, 29, 32, 33). 

This study had some limitations. First, our sample size was relatively small. This was mostly due to the presence of COVID-19; thereby, hospital wards, especially the ICU, had limitations in personnel trafficking, and we could not take more samples. Nevertheless, the limitations of most investigations are related to the small number of studied isolates in different countries (5). Moreover, we did not evaluate other resistance mechanisms in CRE isolates because it was beyond the scope of this work. 

**Table 1:** The number of carbapenem-resistant Enterobacteriaceae (CRE) isolates acquired from inpatients in different days

	Days of hospitalization		
CRE acquired from patients	I	III	VII
		* *	* *
	* * * * * * * *		
	* * * *	* * * *	

**Table 2:** Risk factors associated with carbapenem-resistant Enterobacteriaceae (CRE) fecal carriage among inpatients

Patient characteristics	CRE carriers n = 14* (%)	non- CRE carriers n=33 (%)	*P*-value
Age			
25-35 36-45 46-55 56-65 66-75 76-85 86-95	1 (7.1) 1 (7.1) 3 (21.4) 3 (21.4) 3 (21.4) 2 (14.2) 1 (7.1)	3 (9.1) 4 (12.1) 7 (21.2) 6 (18.1) 7 (21.2) 4 (12.1) 2 (6)	0.367
Sex			
Male Female	6 (42.8) 8 (57.2)	23 (69.7) 10 (30.3)	0.108
Admission within 3 months	8 (57.2)	9 (27.2)	0.095
Antibiotics use within 3 months	12 (85.7)	21 (63.6)	0.175
Catheter Central Venous Line	5 (35.7)	9 (27.2)	0.729
Immunosuppressor drugs use	5 (35.7)	4 (12.1)	0.102
Chemotherapy	2 (14.2)	4 (12.1)	1.00
Mechanical ventilation	12 (85.7)	27 (81.8)	1.00
Surgery	6 (42.8)	8 (24.2)	0.297
Malignancy	6 (42.8)	10 (30.3)	0.506
Autoimmune disease	1 (7.1)	4 (12.1)	1.00
Diabetes mellitus	4 (28.5)	6 (18.1)	0.456
Urinary catheter	10 (71.4)	22 (66.6)	1.00

*14 isolates recovered from 13 patients with different molecular typing

**Table 3:** Antibiotic susceptibility pattern of carbapenem-resistant Enterobacteriaceae (CRE) isolates

Antibiotic	Total (N=21) No. (%)	
	Resistant	Susceptible
Ampicillin	19 (90.5)	2 (9.5)
Ciprofloxacin	18 (85.7)	3 (14.3)
Ampicillin	17 (80.95)	4 (19.05)
Gentamicin	13 (61.9)	8 (38.1)
Amikacin	20 (95.2)	1 (4.8)
Cefoxitin	21 (100)	0 (0)
Aztreonam	21 (100)	0 (0)
Ceftazidime	12 (57.1)	9 (42.9)
Piperacillin/tazobactam	19 (90.5)	2 (9.5)
Meropenem	16 (76.2)	5 (23.8)
Imipenem	16 (76.2)	5 (23.8)
Ertapenem	21 (100)	0 (0)

**Table 4:** Antibiotic resistance percentage (%) among carbapenem-resistant Enterobacteriaceae (CRE) isolates according to bacteria

Isolates	ETP	CAZ	AZT	CFO	AMP	MER	CIP	GEN	IMI	AMK	TZP
*K. pneumoniae*	100	100	100	100	100	93.3	93.3	93.3	93.3	80	80
*E. coli*	100	100	100	100	60	80	60	60	40	0	0
*E. aerogenes*	100	100	100	0	100	0	0	0	0	100	0

AMP: Ampicillin; CIP: Ciprofloxacin; GEN: Gentamicin; AMK: Amikacin; CFO: Cefoxitin; AZT: Aztreonam; CAZ: Ceftazidime; TZP: Piperacillin/tazobactam; MER: Meropenem; IMI: Imipenem; ETP: Ertapenem

**Table 5:** Distribution of carbapenemase genes among carbapenemase-producing Enterobacteriaceae (CPE) according to bacteria

CPE	No of isolates (%)	IMP	SME	KPC	GES	VIM	NDM	OXA-48
*E. coli*	5 (25)	0 (0)	0 (0)	0 (0)	0 (0)	2 (40)	0 (0)	4 (80)
* K. pneumoniae*	14 (70)	2 (14)	3 (21)	2 (14)	0 (0)	3 (21)	0 (0)	9 (64)
*E. aerogenes*	1 (5)	0 (0)	0 (0)	0 (0)	0 (0)	0 (0)	1 (100)	0 (0)

C: Cluster; ST: Singleton; CRE: Carbapenem-resistant Enterobacteriaceae; AMP: Ampicillin; CIP: Ciprofloxacin; GEN: Gentamicin; AMK: Amikacin; CFO: Cefoxitin; AZT: Aztreonam; CAZ: Ceftazidime; TZP: Piperacillin/tazobactam; MER: Meropenem; IMI: Imipenem; ETP: Ertapenem

## Conclusion

In summary, our study found an overall prevalence for CRE fecal carriage of 28.2% among hospitalized patients, which was greater than expected from national surveillance data, with 100% MDR phenotype. The present study indicates that OXA-48 is probably the main resistance mechanism in CRE isolates in our region. Early detection and monitoring are suitable and necessary to prevent the further dissemination of these resistant bacteria. To our knowledge, this is the first report documenting the prevalence and risk factors of CRE asymptomatic carriers and infection in patients admitted to the ICU from southern Iran. 

## Authors’ Contributions

RK Conceptualization; ND, RK, BPA, FZ Methodology; ND, VN Analysis of data; ND, VN, RK Data Curation; RK Validation; RK, ND, Investigation; RK, BPA Resources; RK, ND Writing—original draft; RK Writing—review and editing; RK, ND Visualization; RK, BPA Supervision; FZ Clinical advisor; RK, BPA Project administration; RK, BPA Funding acquisition; All authors read and approved the final manuscript. 

## Funding

This work was supported by Shiraz University of Medical Sciences, (Grant number 20441).

## Ethical Approval

This study was in accordance with the institutional Ethics Committee of Shiraz University of Medical Sciences (Approval No. IR.SUMS.REC. 1399.467). 

## Availability of Data and Materials

The data sets used and/or analyzed during the current study are available from the corresponding authors on reasonable request.

## Conflicts of Interest

The authors declare that they have no conflicts of interest.
